# Qualitative Assessment of Local Distribution of Screen for Life Mass Media Materials in Appalachia

**Published:** 2006-03-15

**Authors:** Robin C Vanderpool, Cathy A Coyne

**Affiliations:** University of Kentucky Markey Cancer Control Program; Department of Community Medicine, West Virginia University, Morgantown, WVa

## Abstract

**Introduction:**

*Screen for Life: National Colorectal Cancer Action Campaign *is a multimedia campaign that informs men and women aged 50 and older about the importance of colorectal cancer screening. The Appalachia Cancer Network undertook a qualitative research study to help determine whether *Screen for Life* materials are being used and distributed by organizations serving Appalachian residents and to help assess key informants' perceived acceptability of the materials.

**Methods:**

Semistructured telephone interviews were conducted with 13 state and local informants in three Appalachian states to assess the diversity of community organizations that received the materials, the level of material use, and receptivity to *Screen for Life*.

**Results:**

Regional cancer control programs were more active in promoting *Screen for Life* at local levels than state health departments. Although state health departments are the primary route for distributing *Screen for Life* materials, they did not report the breadth of activities noted by regional cancer control programs. Several local interview respondents were unfamiliar with *Screen for Life,* and respondents who were familiar with *Screen for Life *used the materials in a general, unplanned way. Although some respondents were unfamiliar with the campaign materials, they were interested in *Screen for Life*. No formal evaluations on the effectiveness of the materials were reported.

**Conclusion:**

More guidance on how to implement the *Screen for Life* campaign as a targeted health communication media campaign would be helpful.

## Introduction

### Screen for Life

To increase colorectal cancer screening rates, the Centers for Disease Control and Prevention (CDC), in collaboration with the Center for Medicare and Medicaid Services (CMS) and the National Cancer Institute (NCI), developed a national awareness campaign, *Screen for Life: National Colorectal Cancer Action Campaign* (*Screen for Life*), which was launched in 1999. The campaign messages are based on an extensive literature review, informant interviews, focus groups, conversations with medical experts, and guidance from a professional communications firm (1).

The goal of the campaign is to raise awareness of colorectal cancer screening among all Americans aged 50 years and older; populations of special interest include African Americans, Hispanics, Alaska Natives, and Medicare beneficiaries (1). Components of the *Screen for Life* campaign include television and radio public service announcements (PSAs), posters, brochures, fact sheets, and print advertisements. CDC distributes campaign materials to television, radio, and print media in major market areas throughout the United States. CDC also maintains a Web site that provides additional information about the campaign and instructions for ordering materials (available from www.cdc.gov/screenforlife).

In addition to directly distributing campaign materials, CDC makes campaign materials available to state health departments for distribution to local media. CDC works with intermediaries such as state and local organizations to promote the *Screen for Life* campaign at the local level and increase the likelihood that broadcast media materials are aired at times favorable for reaching target audiences.

According to process evaluation data provided by CDC, *Screen for Life* television PSAs have aired more than 143,000 times in the United States, accounting for 2.2 billion audience impressions and an estimated dollar value of $13 million (CA Gelb, written communication, February 2005). CDC also is monitoring colorectal cancer screening rates using data from the Behavioral Risk Factor Surveillance System and the National Health Interview Survey. Data from both surveys indicate colorectal cancer screening rates are low among U.S. adults aged 50 or older (2,3). However, additional evaluation methods, such as assessing the dissemination of the *Screen for Life* campaign through intermediaries such as state and local organizations — a critical component of cancer prevention and control — have not been examined. It is therefore unknown whether state and local organizations are effectively using this educational resource.

Intermediaries are frequently used to enhance the distribution and use of health communication campaign materials. Intermediaries are individuals or groups that control the flow of information or resources to intended audiences. If intermediaries do not like the messages or materials being distributed, they may serve as barriers to audience exposure (4). When conducting formative research for the design of communication materials, it is important to ask intermediaries to review materials (4); however, it is not always possible for all potential intermediaries to review materials being developed at the national level. It is especially important to examine the role of intermediaries when there is concern that a segment of the target audience is not being reached. Obtaining feedback from intermediaries on the distribution mechanism for the materials and the materials themselves is important when assessing campaign implementation, which is an essential component of process evaluation.

### Colorectal cancer in Appalachia

As defined by the Appalachian Regional Commission (ARC), the Appalachia region encompasses 410 counties in 13 states along the spine of the Appalachian Mountains (Alabama, Georgia, Kentucky, Maryland, Mississippi, New York, North Carolina, Ohio, Pennsylvania, South Carolina, Tennessee, Virginia, and West Virginia) (5). The mortality rate of colorectal cancer in Appalachia (17.2 per 100,000 population) is higher than the national rate (16.9 per 100,000) (6). Of particular interest to this study are the colorectal cancer death rates in Kentucky (18.4 per 100,000), West Virginia (18.2 per 100,000), and Pennsylvania (18.8 per 100,000) (6). Colon and rectal cancer incidence rates in the Appalachian regions of these three states are also reported to be significantly elevated compared with the U.S. rates approximated from NCI's Surveillance, Epidemiology, and End Results (SEER) program (relative risk, colon cancer = 1.13; relative risk, rectal cancer = 1.19) (7). In addition, colorectal screening rates in parts of Appalachia and other rural areas in the United States are lower than national, metropolitan, and suburban rates (2,8). Appalachia is also recognized for growing disparities related to poverty, employment, education, chronic disease and disability, overall health status, health insurance coverage, and access to preventive health care services and medical providers (9,10). Recognizing the need for increased awareness about colorectal cancer screening in Appalachia, the Appalachia Cancer Network (ACN) examined the distribution of *Screen for Life* materials in this region through intermediary organizations, including state health departments and its own program directors. ACN, an NCI-funded Special Populations Network, was created to address cancer health disparities among Appalachian residents in eight states: Kentucky, Maryland, New York, Ohio, Pennsylvania, Tennessee, Virginia, and West Virginia (9). Investigators sought to obtain insight into potential barriers to distributing and using *Screen for Life* materials in Appalachian communities within the network as well as insight into the usefulness of the materials and preferences for the different types of campaign materials. The research question posed by investigators was, Are *Screen for Life* materials being used and distributed by state or local organizations serving Appalachian residents? In addition, the authors examined local intermediaries' perceived acceptability of the materials among Appalachian populations.

## Methods

Semistructured telephone interviews with state and local intermediaries in Kentucky, Pennsylvania, and West Virginia were conducted in February and March 2003 by the Central Highlands ACN Research Coordinator. State-level intermediaries included the three ACN regional program directors and three state health department staff members identified by CDC as being *Screen for Life* state contacts. Using a snowball technique, each state-level intermediary was asked to provide the names of two local contacts to whom he or she distributed *Screen for Life* materials. The purpose of these referrals was to include a broad range of local organizations that received and possibly used the *Screen for Life* materials.

Questionnaire items were related to the 2002 *Screen for Life* campaign materials. The state-level questionnaire inquired about CDC's distribution of the *Screen for Life* materials, how the intermediaries used and disseminated *Screen for Life* materials, whether they found the campaign materials to be effective, and recommendations for improvement. Similarly, the local questionnaire inquired about the distribution and use of *Screen for Life* materials, assessment of the effectiveness of the *Screen for Life* campaign in raising awareness of screening, and recommendations for improvement. Each interview lasted approximately 10 to 15 minutes, and responses were recorded using handwritten notes. The study was approved by the University of Kentucky Institutional Review Board.

## Results

Thirteen interviews were completed; six interviews were completed at the state level and seven at the local level. Local respondents worked in various settings, including local health departments, hospitals, and a state university. One ACN regional office included as a state-level intermediary did not receive the 2002 *Screen for Life* materials from CDC and therefore did not distribute them. However, the office was aware that one of its local partners had used the *Screen for Life* materials and suggested interviewing this partner organization. One state health department identified as an intermediary received the 2002 *Screen for Life* materials but did not distribute them to local organizations because of competing program priorities, a short CDC funding cycle, and staffing limitations. Another state health department received and distributed the *Screen for Life* materials but chose not to contribute names of local organizations to participate in the study.

### Distribution of campaign materials at the state level

The two ACN regions that distributed and used the *Screen for Life* materials reported greater campaign activity than the two state health departments. These two regions worked through their network of field staff and established community coalitions to distribute the *Screen for Life* materials to various settings, including beauty shops, banks, churches, health departments, hospitals, school systems, grocery stores, civic organizations, cancer centers, fitness centers, libraries, senior centers, rural clinics, and cooperative extension offices. As an example, one of the ACN programs distributed 180 posters and more than 8000 brochures to area libraries and 200 posters and 5000 brochures to local Area Agencies on Aging.

The two participating state health departments distributed the *Screen for Life* materials to state employees and local and regional health departments. One state health department disseminated the materials through its six regional cancer prevention coordinators; these coordinators serve in the state's six health districts. The coordinators distributed the brochures at presentations and health fairs throughout the state and promoted the television PSAs. The other state health department made the *Screen for Life* posters available to its local and regional health departments at regional meetings and provided links to CDC's *Screen for Life* Web site through the state health department's listserv. These actions enabled local health departments to access and order materials themselves.

When asked how they would improve CDC's distribution of the *Screen for Life* materials, responses from both ACN and the state health department staff centered on perceived CDC programmatic issues. Comments were made that CDC could work more actively with the states, increase ordering limits, and dedicate more staff for processing orders. It was suggested that materials be given directly to local health departments because, as stated by one respondent, materials were "usually stalled at the state level." This respondent recommended that CDC "target clinics where people are actually being seen and screened."

Rather than commenting on CDC's distribution, one state health department respondent commented on her organization's distribution challenges: "Distribution is an age-old issue. There wasn't enough funding to make sure the health departments used the materials. There was no follow-up. There was no time or money to discover the outcome or to track the use of the materials by the local health departments."

### State-level assessment of campaign materials' effectiveness

The ACN program directors and the state health department staff perceived that the *Screen for Life* materials were successful in stimulating and increasing colorectal cancer screening awareness. However, respondents either were doubtful or unable to answer when asked whether the materials had an effect on improving colorectal cancer screening rates in their communities.

All respondents seemed to recognize *Screen for Life* as an awareness campaign, but as one ACN program director commented, "*Screen for Life* is a great idea. It's good for national awareness, but it needs another level to actually impact colorectal cancer screening." Beyond anecdotal comments from community coalition members, activity reports from ACN field staff, and follow-up surveys with community groups inquiring about future participation in cancer control activities, there were no formal analyses related to material effectiveness.

When asked which of the materials were most effective, several respondents reported that they felt the posters were most effective; one respondent, however, did not feel comfortable answering the question because of a lack of formal evaluation. Other perceptions were related to factors that might affect the effectiveness of the PSAs. For example, PSAs aired late at night may not be as effective as PSAs aired during prime television viewing hours. Another respondent commented, "There just wasn't a reasonable way to get the PSAs to the local health departments and their media contacts. No funding was available to make copies."

### Suggestions for improving the campaign from state-level intermediaries 

The ACN program directors and the state health department staff agreed that a "plan of action" should accompany the *Screen for Life* campaign materials. Each organization using the materials decides how they are used and distributed, but each may not have time to develop an implementation strategy. Study respondents expressed the belief that the *Screen for Life* campaign materials should be part of a comprehensive cancer education program, which would also include successful methods for their use and distribution.

One respondent commented that she especially liked the "Let's Break the Silence" brochure because it was written in a low-literacy format, it was organized into succinct sections, and it included good visuals. She noted, however, that the brochure was not available for the 2003 *Screen for Life* campaign. Respondents offered suggestions, including developing a slide show, using more family-centered rather than individual-focused materials, and creating more materials for health educators. Another respondent stated that enabling the state health department to tag the television PSAs with its own information made the PSAs more personal. 

### Distribution of campaign materials at the local level

As noted previously, local contacts were named by state-level respondents who stated that *Screen for Life* campaign materials had been sent to that individual or organization. Of the seven local contacts, three recalled receiving the *Screen for Life* campaign materials and were familiar with them, two thought "maybe" they had received the materials but were not familiar with *Screen for Life, *and two respondents reported that they had not received the materials nor were they familiar with them. Of the three respondents who received the *Screen for Life* materials and were familiar with them, two used the materials in a similar fashion: both exhibited the posters throughout their communities in clinic waiting rooms, medical examination rooms, physicians' offices, and hospital corridors. Neither respondent targeted a specific audience but rather used a general approach to reach all visitors to these locations. One of these two respondents also used the brochures in community outreach activities, including mobile blood pressure clinics. Neither respondent had assessed how well the materials were received by the audience.

The third community-level respondent who received the *Screen for Life* materials and was familiar with them used the *Screen for Life* materials in an entirely different way. Instead of disseminating the materials, the organization represented by this respondent chose to conduct qualitative research on the materials (11). The organization's staff members reviewed the materials to determine the appropriateness of the materials for Appalachian populations, whether they needed to be tailored to Appalachians, and the best way to disseminate them. The organization conducted focus groups in two Appalachian counties with members of the general public to examine their reactions to the *Screen for Life* brochures and fact sheets. In addition, the organization conducted interviews with the office staff of family practice physicians in the same two counties in which the focus groups were held to determine additional perceptions.

### Local-level assessment of campaign materials' effectiveness

Of the two respondents who used the materials in their clinics, one perceived that the *Screen for Life* campaign was effective in increasing colorectal cancer screening awareness, because it "seems as though patients are doing more screening." The other respondent hesitated when asked this question and ultimately responded with the following comment: "That's a tough question. With awareness campaigns, you need to hit the audience from every angle, especially rural populations. PSAs could have been helpful. We needed something to get their attention, like the American Cancer Society's 'Polyp Man' campaign." The respondent from the organization that conducted focus groups replied, "Honestly, I don't know if *Screen for Life* is effective in raising awareness."

### Suggestions for improving the campaign from local-level intermediaries

Similar to the state-level intermediaries, one community-level respondent recommended training for users of the materials and direction on how to make presentations. Additional suggestions included the development of print-ready materials that could be personalized and tailored to a participating organization, which would "help to save time and keep from reinventing the wheel." One respondent commented, "Couldn't we do more than just handing stuff out? It would be nice to receive a comprehensive program — 'Here's what you do!'" In response to these remarks, particularly the comment about print-ready materials, the study interviewer provided the *Screen for Life* Web site address to the respondent. The respondent went straight to the Web site during the interview and stated, "This is great! I had no idea! It would have been helpful to have known about the *Screen for Life* Web site to download information."

## Discussion

Study results suggest that *Screen for Life* materials were used more when distributed by the ACN through its established coalitions and field staff than when distributed by the state health departments. State health departments, which are CDC's primary route of distribution, did not report the extent of activities or the quantitative distribution described by the ACN programs. It may be advantageous for CDC to conduct a survey of state *Screen for Life* contacts to assess the programmatic issues that may exist in receiving or ordering materials each year. Evaluation results could guide CDC in working more closely with state health department staff to ensure that materials are being distributed and determine the technical assistance that may be needed at the state level. CDC may also want to explore the possibility of collaborating with groups such as NCI's Community Networks Program, which may have additional time, resources, funding, partners, and community outreach mechanisms to actively promote *Screen for Life* materials and messages. In May 2005, NCI funded 25 Community Networks to Reduce Cancer Health Disparities Through Education, Research, and Training, including the new Appalachia Community Cancer Network. The Community Networks Program will address cancer health disparities by conducting community-based participatory education, training, and research among minorities and medically underserved populations (12).

State-level intermediaries did not find the *Screen for Life* materials to be effective in increasing colorectal cancer screening rates in their communities, although no formal evaluations were reported. Respondents suggested that the campaign should be recognized primarily as an awareness campaign, and more needs to be done if screening rates are to be increased. Although respondents were generally satisfied with the materials, it was suggested that more guidance from CDC on how to use the materials would be beneficial. Development of a plan of action or guide to implementation may strengthen the impact of the individual pieces of the *Screen for Life* campaign. Surveying state and community organizations that have successfully used and evaluated the *Screen for Life* campaign materials and publishing a best practices guide may also benefit the *Screen for Life* program.

Although the suggestions provided at the state level are from only six individuals, they represent insightful ways in which the campaign might be modified to assist cancer control program directors in meeting their state needs. Other state- and regional-level program directors are encouraged to share their perceptions of and experiences with *Screen for Life* with CDC so that changes to the campaign meet the needs of agencies disseminating and using the materials.

Based on the results of interviews conducted with community-level intermediaries, *Screen for Life* is not as well-known at the local level as at the state level. Several respondents were not familiar with *Screen for Life*, and respondents who were familiar used the materials in a general, unplanned manner. The respondents perceived the materials to be somewhat effective in raising awareness of colorectal cancer screening, but similar to the state-level respondents, the community-level respondents had not conducted formal evaluations to support their perceptions. Despite the unfamiliarity with *Screen for Life* among some respondents, they were interested in the *Screen for Life* materials and found CDC's Web site to be particularly helpful. Marketing of the *Screen for Life* Web site directly to community health agencies through e-mail, listservs, mailings, presentations, and professional conferences may increase awareness of the information that can be downloaded from the site.

Similar to state-level respondents, community-level respondents reported that organizations need guidance on how to integrate health communication campaign materials into their health care services. Small grants from federal health agencies would allow community-based organizations to use *Screen for Life* materials in a targeted campaign, followed by an evaluation of program effectiveness.

There are several limitations to this study. Because of the subjective nature of qualitative research and the study's small sample size, the findings and statements made throughout the report cannot be generalized to other state- or community-level organizations that conduct *Screen for Life* activities or serve Appalachian or rural populations. Replication of this study with other intermediaries working in other Appalachian or rural areas would help determine whether the results and themes reported here are also found elsewhere.

Although interviews with intermediaries represent a method for collecting detailed information from a variety of individuals, informants may also communicate their own agendas or biases toward the survey's subject matter. In addition, the snowball technique may have excluded other community organizations and other opinions. The use of the telephone to conduct interviews allows relative anonymity, which may result in more frank discussions, but it also prohibits the assessment of nonverbal reactions, and participants may be distracted by their surroundings. Another limitation is possible interviewer bias; the interviewer's knowledge of the study's goals and objectives may have influenced discussions with the respondents. In addition, because the interviews were conducted over the telephone and during one point in time, the true extent of an organization's *Screen for Life* activities may not have been captured. Most telephone calls were made without prior arrangement, which may have resulted in some individuals being unprepared to respond to questions on materials that were sent to them several months previously. Finally, the study was conducted when many state health departments were facing budget shortfalls and prioritizing bioterrorism and homeland security initiatives. Although screening for colorectal cancer is recognized as an important issue, the allocation of time, staff, and financial resources to a screening campaign may not have been deemed imperative.

It was reported to the authors by CDC staff that Appalachian or rural localities were not included in CDC's assessment of colorectal cancer knowledge, behaviors, and screening practices or the testing of *Screen for Life* campaign messages and materials (CA Gelb, oral communication, November 2003). The authors encourage CDC to formally evaluate the appropriateness of *Screen for Life* materials for rural or Appalachian populations, including interviews and focus groups with health care providers and rural and Appalachian residents.

One final interview theme centered on the capacity for colorectal cancer screening among rural or Appalachian populations. Several respondents expressed the belief that many communities may lack the resources for performing colorectal screening tests, such as endoscopy equipment and gastroenterologists. It was also perceived that some patients may not be able to pay for screening. Although raising awareness of colorectal cancer screening is important, barriers such as access to care and financial limitations still need to be addressed, especially in rural and Appalachian populations.
